# Diffuse hepatic hemangioma with single cutaneous hemangioma: an alerting occurrence

**DOI:** 10.1002/ccr3.963

**Published:** 2017-04-24

**Authors:** Faten Al Tasseh, Merna El‐Khansa, Omran Abd, Anoir Abdel Khalek, Nahida El‐Rifai

**Affiliations:** ^1^ Department of PediatricsMakassed General HospitalBeirutLebanon; ^2^ Department of RadiologyMakassed General HospitalBeirutLebanon; ^3^ Dr Anoir Abdel Khalek Center of RadiologyBeirutLebanon

**Keywords:** Consumptive hypothyroidism, cutaneous hemangioma, hepatic hemangioma, infant

## Abstract

Screening of infants with five or more cutaneous infantile hemangiomas with abdominal ultrasound is often recommended. However, diffuse hepatic hemangioma can develop even in patients with single cutaneous hemangioma. This highlights the importance of physical examination and screening ultrasound in these patients.

## Introduction

Liver hemangioma is the most common hepatic vascular anomalies in infancy accounting for 90% of these lesions. They could be multiple in 10–20% of cases 1. Liver hemangioma could be classified as focal, multifocal, and diffuse. Focal hepatic hemangioma (HH) appears as a solitary lesion, multifocal HH had intervening segments of normal hepatic parenchyma, and diffuse HH exhibited innumerable tumors with nearly complete hepatic parenchymal replacement. They are characterized by rapid growth caused by cellular proliferation and spontaneous involution which can be accelerated by the use of angiogenesis inhibitors 2. The liver hemangioma may be asymptomatic or serious complications such as congestive heart failure, consumptive hypothyroidism, and abdominal compartment syndrome can develop 3. Screening with abdominal ultrasonography is often recommended in patients with more than five cutaneous hemangiomas. These infants carry a greater frequency of hepatic hemangioma in comparison with those with fewer than five cutaneous hemangiomas 4.

We present here a 3.5‐month‐old boy infant with single cutaneous hemangioma in whom the diagnosis of diffuse hepatic hemangioma was made because of progressive abdominal distension. Consumptive hypothyroidism was confirmed by laboratory investigations. Propranolol with thyroid replacement therapy was started and was associated with dramatic improvement.

## Case Presentation

H.K.M is a 3.5‐month‐old boy infant was born by cesarean section to a G2P2A0 for failure to progress. The pregnancy was uneventful with no perinatal complications. Birthweight was 3 kg. The physical examination showed a small dome‐shaped hemangioma (1.5 × 1.5 cm) on the right knee. The remaining of the physical examination was unremarkable. The patient was maintained on breastfeeding. At the age of 2 months during regular visit, the patient was noted to have abdominal distension with adequate weight gain. There were no other associated symptoms. The parents were reassured. However, the abdominal distension increased with time so he was referred to our hospital for investigations. Upon admission, the patient was looking pale, active, and alert with no dysmorphic feature. Vital signs were within normal limits for age: weight: 6.3 kg, height: 67 cm, and head circumference: 40 cm. A grade II systolic murmur was heard at the second left intercostal space with active precordium. Chest auscultation was normal. The abdomen was severely distended. The liver was enlarged, firm and nontender with smooth surface. The liver span was 12.5 cm. The spleen was not enlarged. A small hemangioma was noted on the right knee (1.5 × 1.5 cm). The remaining of physical examination was unremarkable. Laboratory investigations revealed hemoglobin of 7.6 g/dL, hematocrit 23, WBC 12.300/mm^3^ (neutrophils 38%, lymphocytes 48%), and MCV 84. Platelets count was 700,000 mm^3^. The absolute reticulocyte count was 92,000/L. BUN, creatinine, electrolytes, calcium, phosphorus, and magnesium were within normal range. The SGOT was 51 U/L, SGPT 18 U/L, GGT 137 U/L, bilirubin total/direct 0.4/0.1 mg/dL. The Alfa fetoprotein level was 9000 IU/L (normal <7 IU/L). TSH level was 220 mU/L (normal 0.7–15.2 mU/L), FT3 3.1 pmol/L (normal 2.6–9.6 pmol/L), and FT4 14.9 pmol/L (normal 11–32 pmol/L).

**Figure 1:**
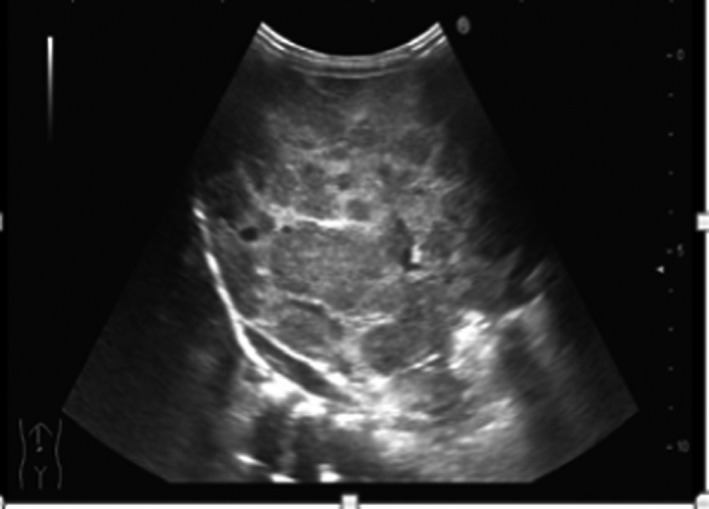
Abdominal ultrasound showing huge diffusely heterogeneous hepatomegaly 13 × 8 ×14.6 cm (volume 974 mL) with well‐delineated innumerable lesions (5–42 mm of diameter) hypoechogenic, some with peripheral halo displacing the adjacent vessels.

**Figure 2:**
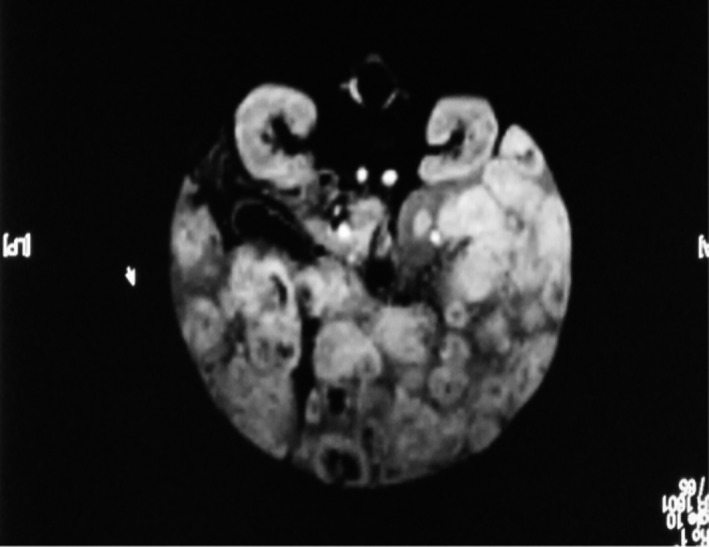
MRI of the abdomen revealing huge heterogeneous hepatomegaly with multiple diffuse intrahepatic well‐defined nodular structures (1–4 cm of diameter) with low signal intensity on T1 and high on T2. There was peripheral enhancement on the arterial phase and homogenization at the delayed venous phase.

Ultrasound of the liver showed diffusely heterogeneous hepatomegaly (13 × 8 ×14.6 cm). There were innumerable lesions from 5 to 42 mm in diameter, well‐delineated hypoechogenic with peripheral halo and echogenic center displacing the adjacent vessels (Fig. 1). Echocardiography was normal. MRI of the abdomen revealed huge heterogeneous hepatomegaly with multiple diffuse intrahepatic well‐defined nodular structures measuring from 1 to 4 cm of diameter with low signal intensity on T1 and high on T2. There was peripheral enhancement on the arterial phase and homogenization at the delayed venous phase compatible with the diagnosis of diffuse liver hemangioma. The hepatic and portal veins were normal. The spleen, kidneys, and the adrenals were normal (Fig. 2).

The patient was started on propranolol 1 mg/kg/day for 3 days then increased to 2 mg/kg/day. No side effects were noted. L‐thyroxin was started at a dose of 25 *μ*g/kg/day. Thyroid function was monitored weekly. Follow‐up after 1 month of treatment showed a reduction in the liver size (liver span 10 cm) with normal TSH level. Ultrasound of the abdomen showed reduction in the liver size with more liver parenchyma seen. Thyroid hormone replacement was stopped after 4.5 months of treatment, and propranolol was continued.

**Figure 3:**
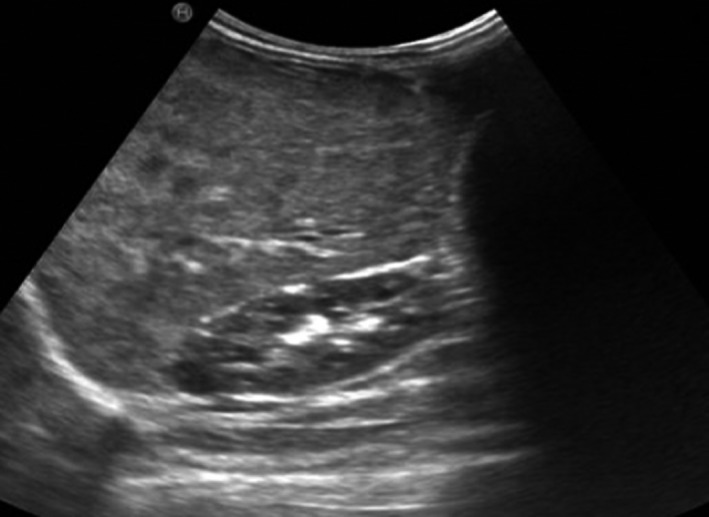
Abdominal ultrasonography showing reduction in liver size and hemangiomas. More liver parenchyma is seen.

Follow‐up at the age of 1 year (8.5 month of treatment) showed marked reduction in the liver size with a liver span of 6.5 cm compared to 12.5 cm at the beginning of treatment with almost complete disappearance of the hemangioma on the right knee. The neurodevelopment of the patient was completely normal. Thyroid hormones level remained normal. The alpha fetoprotein decreased to 5 IU/L. The patient was kept on propranolol 2 mg/Kg/day. Repeat ultrasonography showed further reduction in the liver size and the hemangiomas (Fig. 3).

## Discussion

Screening of infants with five or more cutaneous infantile hemangiomas with abdominal ultrasound is often recommended. Horii et al. found in a prospective multicentric study involving 201 patients with cutaneous hemangiomas an incidence of 16% of hepatic lesions in infants with five or more cutaneous infantile hemangiomas versus 0% in those with fewer than five 4. However, Dickie et al. in retrospective study of 26 patients with hepatic hemangioma found that hepatic hemangioma was seen in patients with none, single, or three cutaneous hemangiomas. This is the reason why they screen any patient with any number of cutaneous hemangioma that present with hepatomegaly with an abdominal ultrasonography 5.

In our patient, the occurrence of diffuse hepatic hemangioma with a single cutaneous hemangioma is alerting. Moreover, the hemangioma was associated with consumptive hypothyroidism that required treatment. Our case in agreement with Dickie et al. highlights the importance of follow‐up physical examination of patient with single cutaneous hemangioma. The development of hepatomegaly should alert the physician to perform abdominal ultrasonography in order not to miss a serious underlying condition such diffuse hepatic hemangioma.

Unlike focal hepatic hemangioma, multifocal and diffuse types may lead to severe complications and possibly death 1, 3. High output cardiac failure (due to aortovenous, aortoportal, and venoportal shunting), Kasabach–Merritt syndrome (due to the intralesional platelet sequestration), anemia, consumptive hypothyroidism, haemoperitonitis (due to spontaneous rupture), and abdominal compartment syndrome can develop 3, 6. Congestive heart failure represents the main cause of mortality in these patients 7. Our patient presented with marked abdominal distension, anemia, elevated α‐FP with normal liver function tests and consumptive hypothyroidism.

Consumptive hypothyroidism is a rare form of hypothyroidism. Huang et al. reported in 2000 the first case of “consumptive hypothyroidism” in a 6‐week‐old infant with infantile hepatic hemangioma. Thyroid hormones were normally synthesized and secreted, but that their rate of degradation was increased. They found an overexpression of D3 and its activity in the hemangioma tissue proportional to the size of the tumor mass, and independent of its localization 8. To date, nineteen cases of consumptive hypothyroidism secondary to hepatic hemangioma were reported in the literature in the pediatric population 9, 10. Our present case is the 20th case. In our case, the TSH level was markedly elevated at the time of diagnosis with normal free T4 and free T3. A high dose of levothyroxine (LT4) 25 *μ*g/kg/day was required at the beginning of the treatment to normalize the TSH level. The TSH level normalized after 1 month of treatment with concomitant reduction in the liver size.

Concerning treatment, a recent meta‐analysis done by Lou et al. in 2013 involving 324 infantile hemangiomas showed that the efficacy of propranolol was greater than other therapies like steroids, interferon, and vincristine in treating this condition with less severe side effects 11. The striking effect of propranolol on infantile hemangioma can be attributed to three molecular mechanisms: vasoconstriction, inhibition of angiogenesis, and induction of apoptosis 12. Hypotension, bradycardia and hypoglycemia are well‐recognized side effects of propranolol and needs to be monitored closely. In our patient, propranolol treatment was well tolerated and was associated with dramatic reduction in the diffuse hepatic hemangioma with subsequent normalization of the thyroid function.

## Conclusion

Although screening of infants with more than five cutaneous hemangiomas is recommended, our case highlights the importance of follow‐up physical examination in patients even with single cutaneous hemangioma. The development of hepatomegaly in such patient should alert the clinician to perform an abdominal ultrasonography in order not to miss a serious associated condition such as diffuse hepatic hemangioma. Combined treatment with propranolol and thyroid hormone replacement are effective in hemangioma reduction and resolution of consumptive hypothyroidism.

## Consent

Written informed consent was obtained from the parents of the patient for publication of this case report.

## Conflict of Interest

All authors have no conflict of interest.

## Authorship

FT and MEK: drafted the initial manuscript and approved the final manuscript as submitted. AO and AAK: participated in the interpretation of imaging studies and radiological follow‐up of the patient and approved the final manuscript as submitted. NER: made the final diagnosis, reviewed the manuscript, and approved the final version as submitted. All authors approved the final manuscript as submitted and agree to be accountable for all aspects of the work.
